# GFPLAIN250m, a global high-resolution dataset of Earth’s floodplains

**DOI:** 10.1038/sdata.2018.309

**Published:** 2019-01-15

**Authors:** F. Nardi, A. Annis, G. Di Baldassarre, E. R. Vivoni, S. Grimaldi

**Affiliations:** 1 Water Resources Research and Documentation Centre (WARREDOC), University for Foreigners of Perugia, Perugia, Italy; 2 Department for Earth Sciences, Uppsala University, Uppsala, Sweden; 3 Centre of Natural Hazards and Disaster Science, CNDS, Uppsala, Sweden; 4 IHE Delft Institute for Water Education, Delft, The Netherlands; 5 School of Earth and Space Exploration, Arizona State University, Tempe, AZ, USA; 6 School of Sustainable Engineering and the Built Environment, Arizona State University, Tempe, AZ, USA; 7 Department for Innovation in Biological, Agro-food and Forest systems (DIBAF), University of Tuscia, Viterbo, Italy; 8 New York University Tandon School of Engineering, Department of Mechanical and Aerospace Engineering, New York City, USA

**Keywords:** Hydrology, Natural hazards

## Abstract

Identifying floodplain boundaries is of paramount importance for earth, environmental and socioeconomic studies addressing riverine risk and resource management. However, to date, a global floodplain delineation using a homogeneous procedure has not been constructed. In this paper, we present the first, comprehensive, high-resolution, gridded dataset of Earth’s floodplains at 250-m resolution (GFPLAIN250m). We use the Shuttle Radar Topography Mission (SRTM) digital terrain model and set of terrain analysis procedures for geomorphic floodplain delineations. The elevation data are processed by a fast geospatial tool for floodplain mapping available for download at https://github.com/fnardi/GFPLAIN. The GFPLAIN250m dataset can support many applications, including flood hazard mapping, habitat restoration, development studies, and the analysis of human-flood interactions. To test the GFPLAIN250m dataset, we perform a consistency analysis with floodplain delineations derived by flood hazard modelling studies in Europe.

## Background & Summary

Floodplains are clearly recognizable from aerial photography by their distinguishable shapes and colors^1^. Riverine areas are not only clearly visible, but are spatially organized following well-known hydrologic and geomorphic properties^2^. Nevertheless, significant uncertainty is associated with existing floodplain delineation methods^3,4^. While floodplain thematic maps are often available, they typically only reflect the context for which they were derived, limiting their broad, multi-sectorial use. For instance, a hydrologic investigation and an aquatic ecology study would likely identify different floodplain extents for the same river corridor depending on the spatiotemporal scale, event or process of interest. To date, a scale-invariant and consistent morphometric zoning of river corridors to identify floodplain landscapes on Earth is still lacking^4^.

The aim of this paper is to present the first global floodplain dataset at 8.33 arcsecond resolution that is equivalent at the equator to a 250-m grid cell size. The GFPLAIN250m dataset is derived implementing a unifying framework for fluvial valley zoning. This framework captures the spatial extent of floodplains by implementing geomorphic algorithms able to identify the alluvium extent as a morphometric descriptor of digital terrain models^5–10^.

The GFPLAIN250m dataset depicts floodplains as unique and identifiable morphological entities that have been primarily shaped by the accumulated effects of geomorphic and hydrologic processes and secondarily by diffusive biotic processes^6,11^. In such a manner, river basins are dissected into domains of low-lying riparian corridors separated from their surrounding landscapes. This scale-invariant, theoretically-consistent representation of the Earth’s floodplains is thus applicable in regions where water-driven erosion and depositional processes govern the morphology of floodplain landscape features. This excludes areas on Earth classified as deserts with low water availability and ice-covered regions with insignificant river flows^12^.

## Methods

### General procedure

The global floodplain map is developed with the GFPLAIN algorithm^6^. Terrain analysis techniques are implemented in GFPLAIN to extract the stream network from a digital terrain model (DTM) of the Earth^13,14^. Each drainage network cell is assigned the maximum potential channel flow depth (*h*) adopting the power law of equation (1) using the contributing area (*A*) as a scaling parameter^15,16^. Equation 1 constitutes an adapted version of the Leopold scaling law^15^ to represent the proportionality, expressed by the ∝ term, between the potential energy associated with floodplain flow shaping process and the river basin morphometric parameter *A*.

(1)h∝Ab

**Figure 1:**
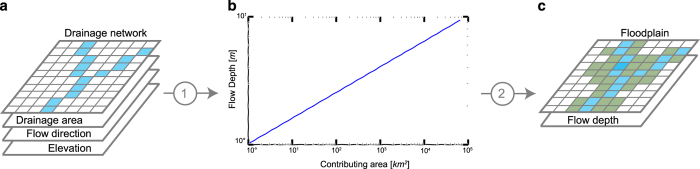
Flow chart describing the DTM analysis and geomorphic scaling law processing for floodplain delineation. Three main steps of the procedure are depicted. (**a**) DTM analysis for flow direction, drainage area and network identification from elevation data. (**b**) Scaling laws implemented for associating a floodplain flow depth to the contributing area of each drainage network grid cell. (**c**) GFPLAIN250m gridded layer is derived by flagging as a floodplain those cells whose elevations are lower than corresponding drainage network flow levels.

The GFPLAIN algorithm^6,17^ produces a gridded floodplain layer by flagging low-lying cells along river corridors. The algorithm recognizes the floodplain extent as formed by those cells, draining to the selected channel location, that are characterized by elevations that are lower than the corresponding maximum channel flow level *H* = *z* + *h*, where *z* is the channel cell elevation obtained from the DTM expressed as absolute elevation in meters above sea level. Figure 1 depicts the three main processing steps of the floodplain identification procedure.

The variation of floodplain flow levels across spatial scales is evaluated by means of the dimensionless *b* exponent^17^ to produce a consistent floodplain zoning analysis (see Technical Validation). The Shuttle Radar Topography Mission (SRTM)^18,19^ DTM, provided by the Consortium for Spatial Information (CGIAR-CSI) at 8.33 arcsecond resolution, covering all regions of the world between −60° and 60° of latitude, is used for floodplain delineations of river basins with a contributing area (*A*) greater than 1000 km^2^. This resolution, equivalent to 250 meters at the equator, is consistent with the spatial scale of other global datasets derived in earth, environmental, social and behavioural science applications for depicting fluvial corridor processes and features^20–22^.

### GFPLAIN algorithm

**Figure 2:**
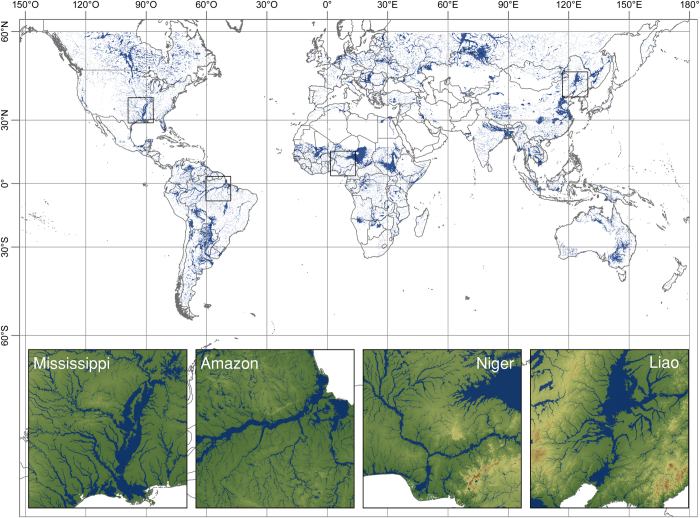
The GFPLAIN250m global floodplain dataset. The GFPLAIN250m is presented in blue color. Insets show floodplains of four major global rivers superimposed on the SRTM dataset.

The GFPLAIN algorithm is organized as a set of Python routines implementing the two main steps of the procedure: (1) Terrain analysis of a DTM for watershed drainage extraction (Fig. 1a), and (2) floodplain delineations (Fig. 1b and c).

The GFPLAIN is a computationally efficient algorithm. Module 2 runs on the order of minutes. Using a standard workstation and the 250-m resolution river network as input, it takes 15 min for delineating the entire floodplains of North and South America. This implies that the largest river basins of the world can be analysed in less than 10 min.

### Code availability

The Python script and user manual of the GFPLAIN algorithm used for generating the GFPLAIN250m dataset are accessible at https://github.com/fnardi/GFPLAIN with instructions for applications and code reuse.

## Data Records

The original SRTM dataset used in this study can be accessed at http://srtm.csi.cgiar.org/ and includes the 250-m SRTM version 4.1 DTM. Figure 2 provides an overview of the dataset, while Table 1 reports a summary of the floodplain mapping for the continents on Earth, except Antarctica.

The GFPLAIN250m dataset can be accessed via figshare (Data Citation 1). Files are stored using both the Esri ASCII raster and the GeoTIFF formats and provided as a seamless dataset using the World Geodetic System 1984 (WGS84) datum and geographic coordinate system. Floodplain raster layers are compressed into a single file zipped for each continent, including the corresponding ASCII or GeoTIFF file. The coding used for each continent and additional information are detailed in the metadata included in the GFPLAIN250m data repository.

## Technical Validation

Evaluation of the quality of the GFPLAIN250m dataset is linked to two main factors: (1) the sources of error and potential uncertainties of the DTM processing for drainage network extraction, and (2) the validation of the geomorphic algorithm for floodplain identification.

The first issue refers to sources of error that impact digital terrain data and known assumptions of DTM analysis techniques for earth science applications. Although it is known that DTM resolution and production method may have a direct impact on the outcomes of the stream network extraction^23–25^, this uncertainty does not propagate to the geomorphic floodplain zoning considering the simulated channel always flows within the fluvial valley^26^. DTM corrections and the use of updated terrain and hydrologic datasets can mitigate this uncertainty^27,28^. Moreover, the potential sources of error of the river network location and profile do not impact the validity of the GFPLAIN250m dataset considering that it is a topographic data descriptor consistent with other morphometric parameters in river basins^20,21^.

**Figure 3:**
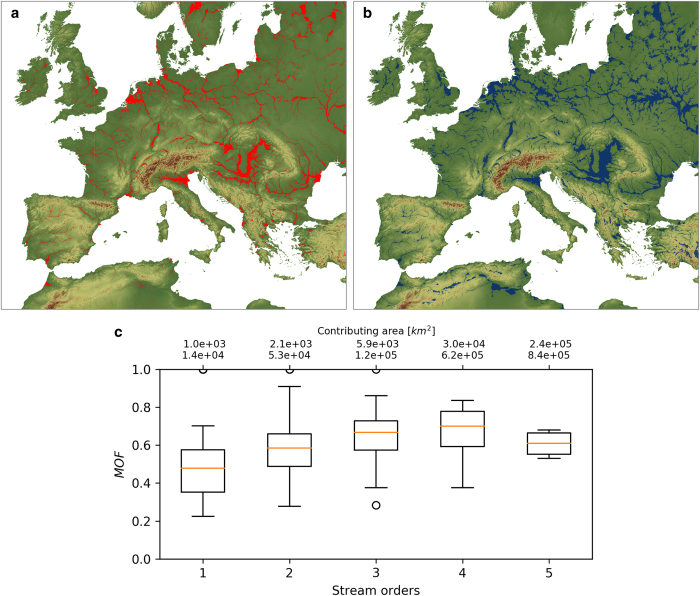
Evaluating the consistency of the geomorphic floodplain model with respect to a flood hazard map. Example of floodplain mapping in Europe using two paradigms. (**a**) Flood hazard event-based mapping using hydraulic simulations of the 200-year synthetic flood design (red color by European Commission, Joint Research Centre). (**b**) Geomorphic floodplain map (blue color). (**c**) Evaluation of the GFPLAIN250m dataset is performed by varying the *b* parameter of the geomorphic scaling law and performing a quantitative comparison with the reference dataset using a measure-of-fit (MOF) index^17^. Box plots represent the statistics of the MOF index obtained by comparing the GFPLAIN250m floodplain zoning with respect to flood hazard zones for European basins of different stream orders.

For the latter, validation of the geomorphic floodplain algorithm is performed by evaluating the outcomes of the GFPLAIN model to varying parameterization of the scaling law. In particular, the sensitivity of results to varying the *b* parameter is investigated. The *b* parameter is varied within a physically feasible range (floodplain flow energy levels within the 10^0^–10^2^ order of magnitude). The optimal *b* is associated to floodplain modelling results that maximize the performances of the geomorphic zoning with respect to a reference floodplain dataset. This consistency analysis is developed by quantifying the effect of *b* value variations on the floodplain zoning behaviour expressed by means of a measure-of-fit index (MOF) based on overlapping, underprediction and overprediction of the floodplain zones^17^. Global fluvial landscape feature zoning is available to depict river channel surface water domains^29,30^. To date, large scale studies delineating floodplain extents using geologic, morphologic or ecologic criteria are not available to benchmark the GFPLAIN250m dataset. Therefore, the 200 years flood prone zoning^31^, based on hydrodynamic models, is used as the only available homogeneous floodplain reference dataset at the global scale^32^. The consistency analysis confirms the validity of the GFPLAIN algorithm in capturing the geomorphic signature of fluvial flooding dynamics. MOF value statistics depict consistent floodplain identification behaviour across the geomorphic, climatic and ecologic diversity of European river basins (Fig. 3). Tests confirm that reasonable ranges of MOF values are obtained, with varying b parameters, supporting the use of a constant parameterization at the global scale with *b* = 0.30. As such, the GFPLAIN250m dataset can be used in combination with global datasets of human settlements, to support large-scale studies of human-flood interactions^32–34^, human pressure on rivers^35^, and changes over time of floodplain and wetland habitats at risk^36–38^. Regional values for the scaling law parametrization can be further refined to capture local variations of geologic, climatic and ecological properties.

## Additional information

**How to cite this article**: Nardi, F. *et al*. GFPLAIN250m, a global high-resolution dataset of Earth’s floodplains. *Sci. Data*. 6:180309 doi: 10.1038/sdata.2018.309 (2019).

**Publisher’s note**: Springer Nature remains neutral with regard to jurisdictional claims in published maps and institutional affiliations.

## Supplementary Material



## Figures and Tables

**Table 1:** The GFPLAIN250m dataset of Earth’s floodplains.

**Continent**	***f***_***p***_ **[km^2^]**	***f***_***pr***_ **[−]**
Europe	806525	0.08
Africa	3853197	0.13
North America	1482914	0.06
South America	2931955	0.16
Asia	3452714	0.08
Oceania	866834	0.10
The floodplain areas are estimated at the continental scale. Estimated variables are: Total floodplain area or *f*_*p*_ [km^2^]; and floodplain area divided by total continental area, the floodplain ratio or *f*_*pr*_ [−].		
